# 

**Published:** 1876-08

**Authors:** 


### BOOKS RECEIVED.

Mémoires sur la Galvonocanstique thermique. Par Le Docteur A. Amussat, Fils, etc., Paris.

Guy's Hospital Reports. Series iii. Vol. XXI. 1876.

Ziemssen's Cyclopaedia of the Practice of Medicine. Vol. XI.

Transactions of the Iowa State Medical Society, 1872 and 1876 inclusive.

---

### PAMPHLETS RECEIVED.

Des Sondes a Demeure et du Conducteur En Baleine. Par Le Docteur A. Amussat.

Seventeenth Annual Announcement of Miami Medical College of Cincinnati, 1876–7.

Twenty-seventh Annual Announcement of the Woman's Medical College of Pennsylvania, Philadelphia, 1876–7.

Thirtieth Announcement of Starling Medical College, etc., Columbus, Ohio, 1876.

Remarks on Urethral Stricture, before the British Medical Association, Aug. 5, 1875. By Fessenden N. Otis, M.D., etc.,Clin. Prof. Genito-Urinary Disease, Col. Physicians and Surgeons of New York. London, 1876.

On Stricture as the Initial cause of Gleet, with remarks on the Urethral Calebre. Being a reply to the paper of Dr. H. B. Sands, on the same subject. By F. N. Otis, M. D., etc., 1876.

Medical Schools and their relation to the Profession. By J. M. Smith, M. D., Charles City, Iowa. 1876.

